# National greenhouse gas budget lays solid foundation for climate action

**DOI:** 10.1093/nsr/nwaf106

**Published:** 2025-03-22

**Authors:** Shilong Piao, Zhengtang Guo

**Affiliations:** Institute of Carbon Neutrality, Sino-French Institute for Earth System Sciences, College of Urban & Environmental Sciences, Peking University, China; State Key Laboratory of Lithospheric and Environmental Coevolution, Institute of Geology and Geophysics, Chinese Academy of Sciences, China

A national greenhouse gas budget, with precision and transparency, is essential for effectively managing and reducing greenhouse gas emissions. It highlights the gap between the current state and the net zero emission target, facilitates scientific planning of mitigation strategies, informs policy makers in formulating climate policies and raises public awareness of the climate footprint of contemporary production and lifestyle. As part of the global stockade under the Paris Agreement, it represents a rare consensus among the international scientific community and all parties of United Nations Framework on Climate Change Convention (UNFCCC). Domestically, it serves to monitor progress towards the net zero target and aids in transitioning to a low-carbon economy, while internationally, it ensures compliance with the Paris Agreement. Therefore, national greenhouse gas budgets are the cornerstone of credible climate action that aims to transform global climate goals from aspirational to achievable.

Despite the consensus on its importance, only a minority of countries regularly report on their national greenhouse gas budgets. Many of these reports suffer from transparency issues in accounting methods, significant delays in reporting, and a lack of verification and precision estimates. Most reports adhere to the lower tiers (tier 1 or tier 2) of the IPCC Guidelines for National Greenhouse Gas Inventories. Although these guidelines have significantly advanced from the 2006 to the 2019 versions, they still fall short in fully accounting for current sources and sinks of greenhouse gases and emissions transfer between nations. Despite rapidly growing reporting infrastructure and modelling capacity, a comprehensive and transparent national greenhouse gas budget remains a major gap. In response to this urgent need, the Global Carbon Project is initiating a new generation of national-scale greenhouse gas budget programs, aligning with the global greenhouse gas budget [1].

With tremendous community resources and efforts, we are delighted to present China's Greenhouse Gas Budget (CNGHG) in this Special Topic, covering sources and sinks of three greenhouse gases (CO_2_, CH_4_ and N_2_O) comprising more than 40 flux terms. The Special Topic includes 2 Research Articles, 2 Reviews and 6 Perspectives, featuring various sectorial, methodological, applicational aspects of China's greenhouse gas budget. Accordingly, China’s net emissions of greenhouse gases in 2023 amounted to 14.3 Gt CO_2_-eq [2], 80% of which was contributed by CO_2_ emissions from the industrial and energy sectors [3]. This budget is verified by atmospheric inversion from measurements of greenhouse gas concentrations [4]. Notably, China's land use is climate-responsible: its intense agricultural greenhouse gas emissions [5] is fully mitigated by greenhouse sinks of natural ecosystems [6,7]. This is in stark contrast to the rest of the world, where land use is a significant net emitter of greenhouse gases. Li *et al.* [8] further discuss a possible forest management solution to maintain China's forest carbon sink. The Perspective papers by Friedlingstein *et al.* [9] and Chevallier and Broquet [10] discuss how both national and global research efforts jointly promote community understanding on ensemble land carbon cycle modelling and atmospheric inversions.

These pioneering efforts of CNGHG provide an open platform for community-based national greenhouse gas accounting and represent a candidate for the best available science practice of greenhouse gas accounting, in supplement to national communications to the UNFCCC. CNGHG provides a gridded and continuous budget that facilitates further research. We anticipate the community will continue the efforts to annually track the national greenhouse gas budget. The paradigm established by CNGHG could also benefit developing countries with expanding measurement infrastructure to obtain measurable, reportable and verifiable national greenhouse gas emissions, thus collectively realizing the shared commitments to address the challenge of climate change.

## References

[1] Canadell JG, Poulter B, Bastos A et al. Natl Sci Rev 2025; 12: nwaf037. 10.1093/nsr/nwaf037 40078373 PMC11900409

[2] Yuan W, Liang M, Gao Y et al. Natl Sci Rev 2025; 12: nwaf069. 10.1093/nsr/nwaf069 40160678 PMC11951099

[3] Lou Z, Zhang H, Zhao X et al. Natl Sci Rev 2025; 12: nwae481. 10.1093/nsr/nwae481 PMC12089771 40396119

[4] Wang Y, Zhang Y, Tian X et al. Natl Sci Rev 2025; 12: nwaf090. 10.1093/nsr/nwaf090 PMC12089751 40396118

[5] Gao Y, Li Z, Hong S et al. Natl Sci Rev 2025; 12: nwaf040. 10.1093/nsr/nwaf040 PMC12089786 40396120

[6] Li T, Liu X, Tian J et al. Natl Sci Rev 2025; 12: nwaf094. 10.1093/nsr/nwaf094 PMC11974395 40196188

[7] Xia J, Xia X, Wang X et al. Natl Sci Rev 2025; 12: nwaf052. 10.1093/nsr/nwaf052 40110031 PMC11921772

[8] Li X, Zhang H, Shang R et al. Natl Sci Rev 2025; 12: nwae464. 10.1093/nsr/nwae464 40060926 PMC11887849

[9] Friedlingstein P, Sitch S, O'Sullivan M. Natl Sci Rev 2025; 12: nwaf073. 10.1093/nsr/nwaf073 40125331 PMC11927394

[10] Chevallier F, Broquet G. Natl Sci Rev 2025; 12: nwaf063. 10.1093/nsr/nwaf063 40125332 PMC11927398

